# Molecular Epidemiology of Helminths at the Wildlife–Livestock Interface in Kazakhstan: Evidence from Sheep and Saiga

**DOI:** 10.3390/pathogens15050550

**Published:** 2026-05-20

**Authors:** Nurzhan Abekeshev, Zhangeldi Ussenov, Rinat Abdrakhmanov, Zukhra Aitpayeva, Marina Svotina, Zhadyra Valiyeva, Askhat Zhumabayev, Albina Darmenova, Ilana Abirova, Zhadyra Ryskaliyeva, Azamat Aitkaliyev, Aigul Kaliyeva, Anargul Berkaliyeva, Rakhima Bissalyyeva, Assylbek Zhanabayev, Gulmira Abulgazimova

**Affiliations:** 1 Institute of Veterinary Medicine and Agrotechnology, Zhangir Khan West Kazakhstan Agrarian-Technical University, Oral 090000, Kazakhstan; 2 Department of Biology, K. Zhubanov Aktobe Regional University, Aktobe 030000, Kazakhstan; 3 Institute of Animal Science and Veterinary Medicine, Saken Seifullin Kazakh Agrotechnical University, Astana 010000, Kazakhstan

**Keywords:** helminth, cross-species transmission, sheep and saiga, Kazakhstan

## Abstract

Helminth infections remain a major constraint to livestock productivity, particularly in regions where domestic animals and wildlife share grazing habitats. This study investigated the molecular diversity and transmission dynamics of helminth communities in sheep (*Ovis aries*) and saiga antelope (*Saiga tatarica*) in West Kazakhstan. A total of 35 animals (20 sheep and 15 saiga) were examined, and helminths were identified using polymerase chain reaction targeting the ITS1 region of ribosomal DNA for nematodes and the mitochondrial cox1 gene for cestodes. Of the 20 analyzed samples, 80% were successfully identified at the molecular level. Detected species included *Haemonchus contortus*, *Trichuris ovis*, *Chabertia ovina*, *Moniezia expansa*, and *Avitellina centripunctata*. Phylogenetic analysis revealed that *Chabertia ovina* isolates from both hosts clustered within a single monophyletic clade, indicating high genetic similarity and supporting potential cross-species transmission. Mitochondrial markers provided higher resolution for cestode differentiation, whereas ITS1 was effective for nematode identification. The predominance of *Chabertia ovina* in saiga suggests ecological adaptation and efficient transmission within wild populations. These findings highlight the epidemiological significance of shared grazing ecosystems and underscore the need for integrated parasite control strategies that consider both livestock and wildlife reservoirs.

## 1. Introduction

Helminth infections remain a major constraint to livestock productivity worldwide, particularly in extensive grazing systems where environmental exposure to infective stages is continuous. Gastrointestinal nematodes and cestodes such as *Haemonchus contortus*, *Trichuris ovis*, *Chabertia ovina*, and *Moniezia expansa* are among the most prevalent parasites affecting small ruminants, leading to reduced weight gain, decreased fertility, and, in severe cases, mortality. The global burden of helminth infections is further exacerbated by increasing anthelmintic resistance, which compromises the effectiveness of conventional control strategies [1,2].

In recent years, increasing attention has been directed toward the role of wildlife in maintaining and disseminating parasitic infections. Wild ungulates can act as reservoirs of helminths, contributing to the persistence of infection in livestock through shared grazing habitats and environmental contamination. The wildlife–livestock interface has therefore emerged as a critical component of parasite epidemiology, particularly in open ecosystems where physical barriers between species are minimal [3,4]. In such systems, cross-species transmission may facilitate the circulation of genetically similar parasite populations, complicating disease control and surveillance efforts.

Central Asia, and particularly Kazakhstan, represents a unique ecological setting characterized by vast steppe landscapes, transhumant livestock production [5,6], and the presence of migratory wild ruminants such as the saiga antelope (*Saiga tatarica*) [7]. Despite the ecological and economic importance of this region, molecular data on helminth diversity and transmission dynamics remain limited. Existing studies in this region rely on morphological identification, which is often insufficient for accurate species differentiation due to phenotypic similarity among closely related taxa and the presence of cryptic species [8,9,10,11]. Consequently, there is a need for molecular approaches to improve taxonomic resolution and support epidemiological investigations.

Molecular genetic tools, particularly polymerase chain reaction (PCR) and DNA sequencing, have become essential for the accurate identification and phylogenetic characterization of helminths. Ribosomal markers such as the internal transcribed spacer 2 (ITS2) region of the nuclear rRNA gene are widely used for nematode identification due to their conserved and variable regions, which enable both broad detection and species-level resolution [1]. In contrast, mitochondrial genes such as cytochrome c oxidase subunit 1 (*cox1*) provide higher discriminatory power for closely related species, particularly among cestodes, due to their elevated mutation rates and maternal inheritance patterns [12]. The integration of these markers allows for robust molecular identification and improved understanding of parasite phylogeny.

Furthermore, molecular epidemiology has increasingly been applied to investigate transmission pathways and host associations. Phylogenetic analyses can reveal patterns of genetic similarity among parasite populations from different hosts, providing indirect evidence of cross-species transmission and shared infection sources [13]. Such approaches are particularly valuable in regions where livestock and wildlife coexist and interact extensively.

Given the limited molecular data on helminths in West Kazakhstan and the potential role of saiga antelope in parasite circulation, this study aimed to (i) identify helminth species infecting sheep and saiga using molecular genetic methods, (ii) analyze their phylogenetic relationships based on ITS1 and *cox1* markers, and (iii) assess the potential for cross-species transmission within shared grazing ecosystems. The findings are expected to contribute to a better understanding of helminth epidemiology in the region and to support the development of evidence-based control strategies.

## 2. Materials and Methods

### 2.1. Animal Ethics

This study was conducted according to the guidelines of the Declaration of Helsinki and approved by the Institutional Review Board and Animal Ethics Committee of the Branch of Kazakh Scientific Research Veterinary Institute (Protocol #2).

### 2.2. Study Area and Sample Collection

The study was conducted in the West Kazakhstan region (51°38′–55°57′ N, 46°9′–46°4′ E), a major livestock-producing area characterized by extensive grazing systems and frequent interactions between domestic and wild ruminants [14]. A total of 35 animals, including 20 sheep (*Ovis aries*) and 15 saiga antelope (*Saiga tatarica*), were examined. These animals were not euthanized specifically for the purposes of this study. The sheep samples were obtained during routine slaughter under standard agricultural practices, while the saiga antelope (*Saiga tatarica*) samples were collected opportunistically in collaboration with local veterinary and wildlife authorities. Information on age and sex was recorded where available and is summarized in Supplementary Table S1. The sheep belonged to local Kazakh breeds typical for the region. Samples were obtained from different anatomical sites, including the small intestine, abomasum, mesentery, liver, and large intestine, across animals of varying age and sex groups. Sampling locations included several districts (Karatobe, Kaztalov, Baiterek), representing a geographically diverse population. The taxonomic identification of the recovered parasites based on their morphological characteristics was carried out using established diagnostic guides and reference atlases [15,16]. Helminths were collected directly from host organs during necropsy, allowing recovery of intact specimens for subsequent morphological and molecular characterization. Individual parasites were carefully isolated and processed separately for downstream analyses. DNA extraction and PCR amplification were performed on single-specimen isolates rather than on fecal material, thereby eliminating the risk of cross-reactivity in cases of mixed infections.

### 2.3. DNA Manipulation and PCR Amplification

Genomic DNA was extracted from helminth specimens using the QIAamp Fast DNA Stool Mini Kit (QIAGEN, Hilden, Germany), following the manufacturer’s instructions. DNA quality and concentration were assessed prior to downstream applications.

Molecular identification was performed according to the protocol described by Deplazes, van Knapen [17]. In brief, for nematodes, the internal transcribed spacer 1 (ITS1) region of the 18S rRNA gene was amplified using the primer NC13/NC2, the forward primer 5′-ATCGATGAAGAACGCAGC-3′, and the reverse primer 5′-TTAGTTTCTTTTCCTCCGCT-3′. For cestodes, a fragment of the mitochondrial cytochrome c oxidase subunit 1 (*cox1*) gene was amplified using the primer JB3/JB4.5, the forward primer 5′-TTTTTTGGGCATCCTGAGGTTTAT-3′, and the reverse primer 5′-TAAAGAAAGAACATAATGAAAATG-3′. Each reaction contained 10 pmol of each primer and approximately 100 ng/µL of template DNA.

PCR amplification was performed in a total reaction volume of 25 μL, containing 12.5 μL of 2× PCR master mix (including Taq DNA polymerase, reaction buffer, MgCl_2_, and dNTPs), 0.5 μL of each primer (10 pmol), 1 μL of template DNA, and nuclease-free water to volume. The conditions for DNA amplification were as follows: initial denaturation at 95 °C for 5 min, followed by 35 cycles of denaturation at 94 °C for 30 s, annealing at 55 °C for 30 s, elongation at 72 °C for 30 s, and a final extension at 72 °C for 7 min. PCR products were separated by electrophoresis in 1% agarose gel using 1× TAE buffer. Visualization was performed under UV illumination. Each PCR run included both positive and negative controls. Positive controls consisted of previously confirmed helminth DNA samples, while negative controls contained nuclease-free water.

### 2.4. DNA Sequencing and Phylogenetic Analysis

Amplified products were purified and sequenced according to the method published by Bowles, Blair [18] using BigDye Terminator chemistry on an ABI 3130XL Genetic Analyzer (Applied Biosystems, Foster City, CA, USA). Raw sequences were edited and assembled using BioEdit v7.0.

Phylogenetic relationships were inferred using nucleotide sequences of ITS1 and *cox1* genes. Sequences obtained in this study were aligned with reference sequences retrieved from GenBank. Phylogenetic trees were constructed using standard algorithms (Neighbor-Joining or Maximum Likelihood), and node support was evaluated using bootstrap analysis (1000 replicates).

## 3. Results

### 3.1. Molecular Identification of Helminths

Helminth infections were detected in 20 out of 35 examined animals (57.1%). Among host species, 7 of 20 sheep (35.0%) and 13 of 15 saiga antelope (86.7%) were positive for helminths. Of the 20 helminth-positive samples, 16 (80.0%) were successfully amplified and identified by molecular methods, while 4 samples (20.0%) failed to yield usable sequences, likely due to DNA degradation or insufficient template quality. Molecular analysis revealed the presence of both nematodes and cestodes in sheep and saiga populations. The identified species included *Moniezia expansa*, *Avitellina centripunctata*, *Haemonchus contortus*, *Trichuris ovis*, and *Chabertia ovina*. PCR amplification and sequencing confirmed the taxonomic identity of the recovered helminths. The obtained nucleotide sequences were deposited in the NCBI GenBank (Supplementary Table S2). Specifically, ITS1 sequences of nematodes (*Haemonchus contortus*, *Trichuris ovis*, *Chabertia ovina*) were assigned accession numbers (PZ309116, PZ309117, PZ309118, PZ309119, PZ309120, PZ309121, PZ309122, PZ309123, PZ309124, PZ309125, PZ309126, PZ309127), while mitochondrial cox1 sequences of cestodes (*Moniezia expansa*, *Avitellina centripunctata*) were deposited under accession numbers (PZ309129, PZ309130, PZ309131).

These findings demonstrate a diverse helminth community circulating among domestic and wild ruminants. In sheep, infections were primarily associated with *Moniezia expansa* (2/20), *Haemonchus contortus* (2/20), *Trichuris ovis* (1/20), and *Chabertia ovina* (1/20), with localization mainly in the small intestine and abomasum. In saiga antelope, *Chabertia ovina* was the most frequently detected species, identified in (9/20) samples (45%)**,** while *Avitellina centripunctata* was identified in a single case. The high prevalence of *Chabertia ovina* in saiga samples (90%) suggests potential adaptation or efficient transmission within wild populations.

### 3.2. Phylogeny

**Figure 1:**
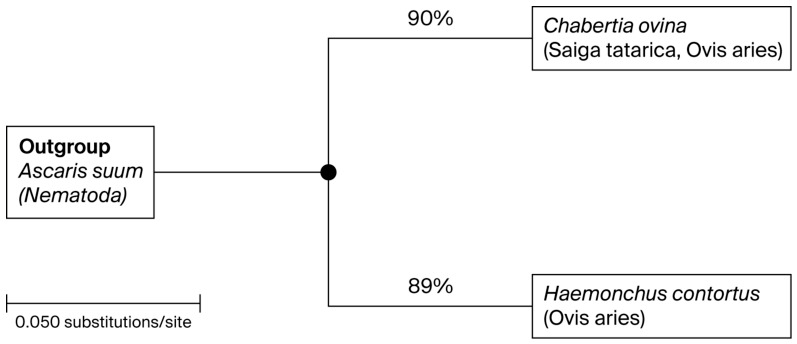
Phylogenetic tree based on nucleotide sequences of the ITS1 region of ribosomal DNA, illustrating the relationships among nematodes isolated from *Ovis aries* and *Saiga tatarica*. The tree was constructed using the Neighbor-Joining method. Bootstrap values (%) are indicated at the nodes and were calculated based on 1000 replicates. Only nodes with bootstrap support ≥75% are shown, while nodes with lower support values were collapsed to improve clarity and the reliability of phylogenetic interpretation. The scale bar represents the number of nucleotide substitutions per site. *Ascaris suum* was used as an outgroup to root the tree.

Phylogenetic analysis based on ITS1 sequences demonstrated that isolates of *Chabertia ovina* from sheep and saiga clustered within a single monophyletic clade together with reference sequences from GenBank (EU086374, EU086375, PQ394620), with strong bootstrap support (~90%). The absence of host-specific clustering indicates a high degree of genetic similarity and suggests possible cross-species transmission. Similarly, *Haemonchus contortus* isolates formed a well-supported clade (~89%), confirming accurate species identification. *Trichuris ovis* sequences formed a distinct and more divergent lineage, reflecting their taxonomic separation from Strongylida nematodes (Figure 1).

**Figure 2:**
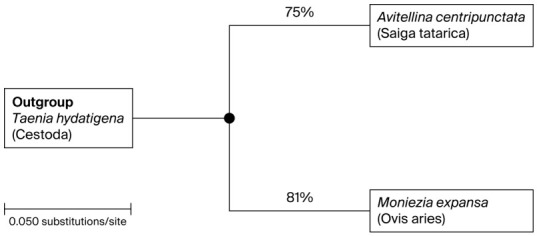
Phylogenetic tree based on nucleotide sequences of the mitochondrial *cox1* gene, illustrating the relationships between the cestodes *Avitellina centripunctata* and *Moniezia expansa*. The tree was constructed using the Neighbor-Joining method. Bootstrap values (%) are indicated at the nodes and were calculated based on 1000 replicates. For clarity, only nodes with bootstrap support ≥75% are shown, while branches with lower support were collapsed. The scale bar represents the number of nucleotide substitutions per site. *Taenia hydatigena* was used as an outgroup to root the tree.

Phylogenetic analysis using the mitochondrial *cox1* gene revealed clear separation between cestode species (Figure 2). *Avitellina centripunctata* isolates clustered with reference sequences, forming a monophyletic clade with moderate bootstrap support (75%), indicating intra-species variability. *Moniezia expansa* isolates formed a distinct, well-supported clade (81%), confirming species identity and demonstrating the higher resolution of mitochondrial markers compared to ribosomal genes. The clustering of *Chabertia ovina* isolates from both sheep and saiga within a single genetic lineage suggests active circulation of this parasite between domestic and wild hosts. This finding highlights the epidemiological significance of shared grazing ecosystems and supports the hypothesis of interspecies transmission.

### 3.3. Morphological Characterization of Recovered Helminths

**Figure 3:**
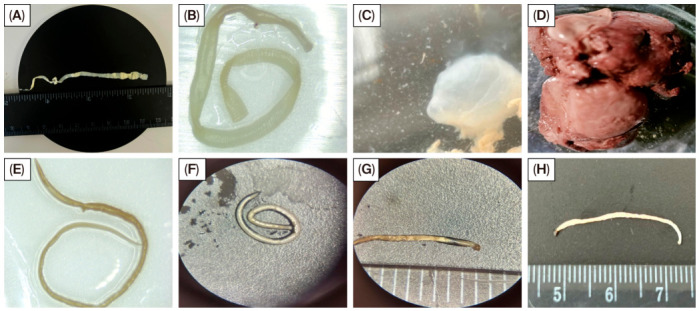
Representative morphological features of helminths recovered from sheep (*Ovis aries*) and saiga (*Saiga tatarica*) in West Kazakhstan. (**A**) Adult cestode specimen showing segmented strobila (proglottids) with scale reference; (**B**) cestode fragments demonstrating characteristic segmentation; (**C**) larval cestode stage observed within host tissue; (**D**) macroscopic tissue lesion associated with parasitic infection; (**E**–**H**) strongylid nematodes under light microscopy, displaying typical elongated cylindrical morphology, including coiled (**F**) and extended (**H**) forms, as well as body structure (**G**). All morphological features are consistent with standard taxonomic descriptions and support molecular identification results.

Macroscopic and microscopic examination of the recovered helminths revealed morphological features consistent with established diagnostic criteria (Figure 3). Adult cestodes exhibited a typical segmented strobila composed of distinct proglottids, with elongated ribbon-like bodies readily visible to the naked eye. In some specimens, larval cestode stages were observed within host tissues as rounded, translucent cyst-like structures.

## 4. Discussion

This study provides molecular evidence of a diverse helminth community circulating among domestic sheep (*Ovis aries*) and saiga antelope (*Saiga tatarica*) in West Kazakhstan. The identification of key gastrointestinal nematodes (*Haemonchus contortus*, *Trichuris ovis*, *Chabertia ovina*) and cestodes (*Moniezia expansa*, *Avitellina centripunctata*) highlights the complexity of parasite assemblages in mixed grazing ecosystems. Similar patterns of high parasite diversity in extensive grazing systems have been reported globally, where environmental contamination and host overlap facilitate transmission [19]. The predominance of *Chabertia ovina* in saiga populations suggests ecological adaptation and efficient transmission under steppe conditions. Comparable findings have been described in wild ungulates, where gastrointestinal nematodes persist due to continuous exposure and lack of intervention [20,21].

Sequence analysis of the amplified ITS1 and *cox1* regions revealed a high degree of similarity with corresponding reference sequences available in GenBank (EU086374, EU086375, PQ394620). Minor nucleotide variations were observed among isolates; however, these differences did not affect species-level identification and did not result in the formation of distinct phylogenetic clusters. No unique or diagnostic mutations indicative of novel lineages were detected within the analyzed dataset. The combined use of ITS1 (18S rRNA) and mitochondrial *cox1* markers enabled reliable species-level identification with an overall success rate of 80%. ITS1 proved effective for nematode identification, although its limited phylogenetic resolution for deeper divergence is consistent with previous studies [12]. In contrast, the mitochondrial *cox1* gene demonstrated superior discriminatory power, allowing clear differentiation among cestode species. This aligns with growing evidence that mitochondrial markers provide higher resolution due to increased mutation rates and reduced conservation [12].

A key finding of this study is the absence of host-specific clustering in *Chabertia ovina*, with isolates from sheep and saiga forming a single monophyletic clade. This strongly supports active cross-species transmission. Such transmission dynamics are widely documented in systems where wildlife and livestock share grazing areas. For example, studies have shown that wild ungulates act as reservoirs, maintaining parasite populations and facilitating spillover into domestic herds [22,23]. The ecological context of West Kazakhstan, characterized by open steppe landscapes and overlapping grazing, further supports this mechanism [14,24,25]. This implies the likelihood of cross-transmission of parasites between different host species. Similar epidemiological patterns have been reported in Central Asian and African pastoral systems [11,26]. The circulation of helminths between sheep and saiga has important implications for animal health and productivity. *Haemonchus contortus*, in particular, is one of the most pathogenic nematodes in small ruminants, causing anemia and production losses [27]. Shared parasite populations may also contribute to the persistence of infection despite control measures [28] and increase the risk of anthelmintic resistance, a growing global concern [29]. These findings highlight the necessity of integrated parasite management strategies that incorporate both livestock and wildlife reservoirs.

An additional contextual factor relevant to the interpretation of our findings is the use of anthelmintic treatments in the region. Although detailed farm-level treatment records specific to West Kazakhstan remain limited, studies from Kazakhstan indicate the use of broad-spectrum antiparasitic formulations in sheep, including albendazole- and ivermectin-based regimens [4]. In addition, studies conducted in West Kazakhstan have evaluated albendazole-based treatment approaches in saiga antelope, confirming the regional relevance of anthelmintic interventions in wild populations [30]. Recent investigations have also documented substantial overlap in helminth fauna between saiga and sheep in western Kazakhstan, supporting the existence of a shared parasite pool at the wildlife–livestock interface [7].

This study has several limitations that should be considered when interpreting the findings. First, the sample size was relatively small (*n* = 35), which limits the statistical power and generalizability of the results. Consequently, formal statistical analyses comparing infection prevalence between host species or regions were not performed, as the sample size and uneven distribution of identified specimens would not support robust inferential testing. Second, sampling was conducted at a single time point, precluding assessment of seasonal dynamics in helminth transmission. Third, molecular identification was successful in 80% of samples, potentially introducing bias due to incomplete amplification or sequencing failures. Additionally, the molecular analysis was based on single genetic markers (ITS1 and cox1), which are suitable for species identification but provide limited resolution for assessing broader genetic diversity. Consequently, additional sources of variation present in other genomic regions may not be captured. Finally, ecological drivers of transmission, such as grazing overlap and environmental contamination, were not quantitatively assessed.

In conclusion, our study demonstrates a diverse helminth community shared between sheep and saiga in West Kazakhstan, emphasizing the epidemiological importance of the wildlife–livestock interface. The lack of host-specific clustering, particularly for *Chabertia ovina*, supports ongoing cross-species transmission within shared grazing systems. The combined use of ITS1 and cox1 markers enabled reliable molecular identification. These findings highlight the need for integrated parasite control strategies that incorporate wildlife reservoirs. Future longitudinal and genomic studies are required to better resolve transmission dynamics and inform sustainable control measures.

## Data Availability

The data that support the findings of this study are available from the corresponding author upon reasonable request.

## Supplementary Materials

The following supporting information can be downloaded at: https://www.mdpi.com/article/10.3390/pathogens15050550/s1, Table S1: Helminths detected in sheep and saiga of different sex and age groups in the West Kazakhstan region; Table S2: Nucleotide sequences and NCBI GenBank accession numbers of the studied helminth species; Figure S1: Agarose gel electrophoresis of PCR products targeting the ribosomal ITS1 region (NC13/NC2 primers) in nematode samples and the mitochondrial *cox1* gene in cestode samples.
