# Supplementary material for: Molecular Epidemiology of Helminths at the Wildlife–Livestock Interface in Kazakhstan: Evidence from Sheep and Saiga
Source: Pathogens. 2026 May 20;15(5):550. doi: 10.3390/pathogens15050550 (PMC13209991; doi:10.3390/pathogens15050550)

Table S1. Helminths detected in sheep and saiga of different sex and age groups in the West Kazakhstan region

No.	Localization	Age and Sex	Sampling Location	Genetic Identification
Helminths in sheep				
1	Small intestine	1.7 months, female	Karatobe	*Moniezia expansa*
2	Mesentery	2 years, female	Baiterek	Negative
3	Abomasum	2.5 months, female	Karatobe	*Haemonchus contortus*
4	Small intestine	2 years, female	Akzhaik	Negative
5	Small intestine	3 years, female	Syrym	*Haemonchus contortus*
6	Small intestine	5 years, female	Terekti	*Moniezia expansa*
7	Small intestine	3 months, female	Karatobe	*Trichuris ovis*
8	Large intestine	2.5 months, female	Karatobe	*Chabertia ovina*
Helminths in saiga				
9	Small intestine	4 months, male	Taskala	*Avitellina centripunctata*
10	Liver	5 years, female	Kaztalov	Negative
11	Small intestine	4 years, female	Kaztalov	*Chabertia ovina*
12	Small intestine	4 years, female	Kaztalov	*Chabertia ovina*
13	Small intestine	1 year, female	Kaztalov	Negative
14	Small intestine	5 months, male	Kaztalov	*Chabertia ovina*
15	Small intestine	2 years, female	Kaztalov	*Chabertia ovina*
16	Small intestine	2 years, female	Kaztalov	*Chabertia ovina*
17	Small intestine	2 years, female	Kaztalov	*Chabertia ovina*
18	Small intestine	3 years, female	Kaztalov	*Chabertia ovina*
19	Small intestine	4 years, female	Kaztalov	*Chabertia ovina*
20	Small intestine	2 years, female	Kaztalov	*Chabertia ovina*

Table S2. Nucleotide sequences and NCBI GenBank accession numbers of the studied helminths.

**№**	**Animals**	**Gene region**	**Identified helminths**	**Accession numbers:**
1	*Ovis aries*	*Cox1*	*Moniezia expansa*	PZ309129
2	*Ovis aries*	*ITS 1 NC13/NC2*	*Haemonchus contortus*	PZ309116
3	*Ovis aries*	*ITS 1 NC13/NC2*	*Haemonchus contortus*	PZ309117
4	*Ovis aries*	*Cox1*	*Moniezia expansa*	PZ309130
5	*Ovis aries*	*ITS 1 NC13/NC2*	*Trichuris ovis*	PZ309118
6	*Ovis aries*	*ITS 1 NC13/NC2*	*Chabertia ovina*	PZ309119
7	*Saiga tatarica*	*Cox1*	*Avitellina centripunctata*	PZ309131
8	*Saiga tatarica*	*ITS 1 NC13/NC2*	*Chabertia ovina*	PZ309120
9	*Saiga tatarica*	*ITS 1 NC13/NC2*	*Chabertia ovina*	PZ309121
10	*Saiga tatarica*	*ITS 1 NC13/NC2*	*Chabertia ovina*	PZ309122
11	*Saiga tatarica*	*ITS 1 NC13/NC2*	*Chabertia ovina*	PZ309123
12	*Saiga tatarica*	*ITS 1 NC13/NC2*	*Chabertia ovina*	PZ309124
13	*Saiga tatarica*	*ITS 1 NC13/NC2*	*Chabertia ovina*	PZ309125
14	*Saiga tatarica*	*ITS 1 NC13/NC2*	*Chabertia ovina*	PZ309126
15	*Saiga tatarica*	*ITS 1 NC13/NC2*	*Chabertia ovina*	PZ309127
16	*Saiga tatarica*	*ITS 1 NC13/NC2*	*Chabertia ovina*	PZ309128

Figure S1. (a) Agarose gel electrophoresis of PCR products targeting the ribosomal ITS1 region (NC13/NC2 primers) in nematode samples. (b) Agarose gel electrophoresis of PCR products targeting the mitochondrial *cox1* gene in cestode samples. The first five samples correspond to the same study, while the remaining two samples 6 and 7 are replicates (Samples 2 and 6 in the left figure). K+ denotes the positive control, and K− denotes the negative control (dH_2O). Electrophoresis was performed in a 1% agarose gel using 1× TAE buffer.

a)

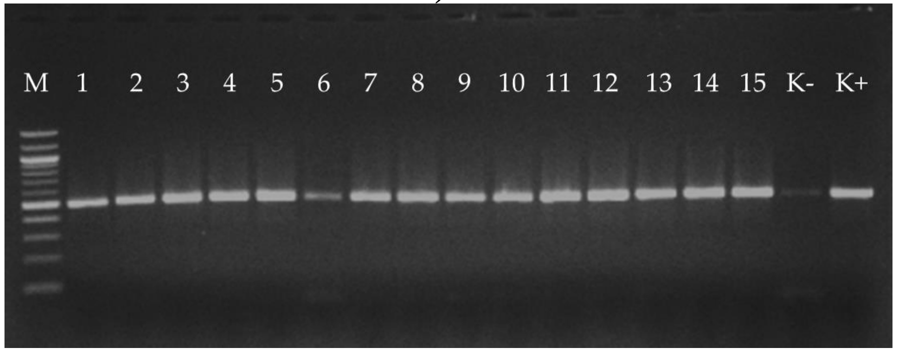

b)

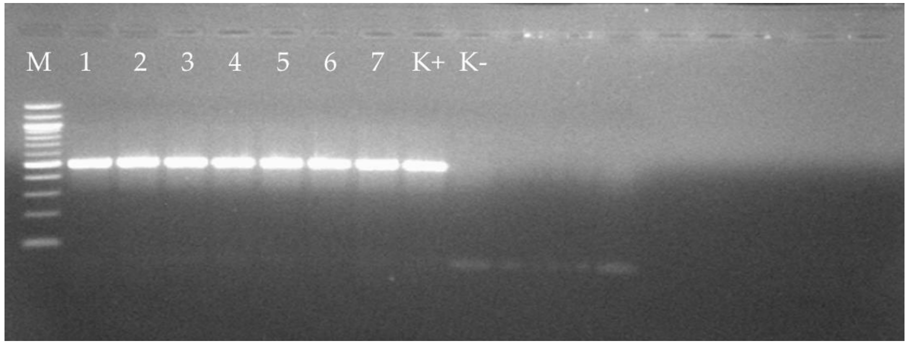

Supplement: Supplementary file 1 [file pathogens-15-00550-s001.zip › pathogens-4283219-updated supplementary.pdf]
